# On the Febrifuge Substance Discovered in the Peruvian Bark

**Published:** 1804-11-01

**Authors:** 


### On the Febrifuge Substance discovered in the Peruvian Bark.

THE celebrated chemist, Mr. Seguin, has read several memoirs before the National Institute, concerning his experiments for discovering the febrifuge principle in the Peruvian bark, its nature, and the quantity which is contained in the different sorts of that medicament. The exterior appearance and taste have hitherto been considered as the only means by which the goodness of that drug is judged, which, however, but imperfectly indicate the presence and quantity of the true febrifuge principle. With a view of ascertaining the quality of the Peruvian Bark in this respect, Mr. Seguin examined the constituents of this medicinal body separately, and also its chemical properties; by which means he succeeded in discovering very material differences in the febrifuge principle of the bark, from the other constituent particles. The chief characteristic of this substance is, *that it precipitates the solution of tanin, but not the solution of glue and of sulphat of iron.* When these marks are not perceived in the Peruvian Bark, it is a proof of its being either adulterated or entirely deprived of the febrifuge principle. The author having examined all the different sorts of Peruvian Bark was induced to conclude, that only a small quantity of pure bark is brought to us, as by far the greatest part of bark that is imported into Europe, possesses very little of the febrifuge matter; matter; it is either of an inferior quality or not at all present in the common sorts of bark. These results are very important, as the Peruvian Bark proves efficacious in diseases only in proportion to the febrifuge principle it contains, and such barks as are deprived of it are rather prejudical than useful to the constitution. This observation likewise induced Mr. Seguin to use his endeavours to find out a febrifuge substance which might be safe, efficacious, accommodated to the constitution, and at the same time so cheap, that no advantage could be derived from adulterating it. With this view he examined the true cause of fever, and the nature of the febrifuge principle contained in the Peruvian Bark, and in what manner it acted on the body. In consequence of his experiments and enquiries, he recommends the *glue or animal jelly, evaporated to dryness,* as a new febrifuge remedy, which unites in itself all the medicinal as well as economical advantages that may be expected.——This substance, therefore, has been tried in a series of cases, particularly by some Italian physicians, and is become a new matter of investigation amongst the physicians on the continent. A publication on this subject has been edited by Dr. *Gautieri,* in which a very favourable account is given of the animal jelly as a substitute for the Peruvian bark.

